# Blurring contact maps of thousands of proteins: what we can learn by reconstructing 3D structure

**DOI:** 10.1186/1756-0381-4-1

**Published:** 2011-01-13

**Authors:** Marco Vassura, Pietro Di Lena, Luciano Margara, Maria Mirto, Giovanni Aloisio, Piero Fariselli, Rita Casadio

**Affiliations:** 1 Department of Computer Science, University of Bologna, Bologna, Italy; 2 Lecce & SPACI Consortium, Department of Innovation Engineering, University of Salento, Lecce, Italy; 3 Biocomputing Group, Department of Biology, University of Bologna, Bologna, Italy

## Abstract

**Background:**

The present knowledge of protein structures at atomic level derives from some 60,000 molecules. Yet the exponential ever growing set of hypothetical protein sequences comprises some 10 million chains and this makes the problem of protein structure prediction one of the challenging goals of bioinformatics. In this context, the protein representation with contact maps is an intermediate step of fold recognition and constitutes the input of contact map predictors. However contact map representations require fast and reliable methods to reconstruct the specific folding of the protein backbone.

**Methods:**

In this paper, by adopting a GRID technology, our algorithm for 3D reconstruction FT-COMAR is benchmarked on a huge set of non redundant proteins (1716) taking random noise into consideration and this makes our computation the largest ever performed for the task at hand.

**Results:**

We can observe the effects of introducing random noise on 3D reconstruction and derive some considerations useful for future implementations. The dimension of the protein set allows also statistical considerations after grouping per SCOP structural classes.

**Conclusions:**

All together our data indicate that the quality of 3D reconstruction is unaffected by deleting up to an average 75% of the real contacts while only few percentage of randomly generated contacts in place of non-contacts are sufficient to hamper 3D reconstruction.

## Background

A major problem of the genomic era is how to link the protein sequence to the protein structural and functional space. When no template with high sequence homology to the target is found in the Protein Data Base (PDB), then building by homology cannot be safely applied. In these cases the protein structure can be predicted with ab initio methods whose scoring capability is poor when no conserved structural domain is recognized in the target. Structural features, including structural conserved domains, disulfide bonds, protein secondary structure, residue solvent accessibility and how residues contribute to local stability (contact residues), can to some extent help in constraining the protein 3D structure. Residues are defined to be in contact in the protein structure when they interact within a fixed distance (threshold) that is routinely set at a value ≥ 7 Å. Residue contact prediction was exploited with different approaches, including statistical and probabilistic methods [1]. In a contact map representation of the protein 3D structure, all the short and long range interactions promoting protein stability emerge to different extent depending on the threshold value adopted to compute the 2D projection. However, this representation poses first of all the problem of structure reconstruction. Recently it has been shown that the problem of computing a set of 3D coordinates consistent with some given contact map is equivalent to the unit-disk-graph realization, which is NP-hard [2]. Other well studied similar problems are structure determination from NMR data [3,4] and protein conformational freedom [5]. However the different solutions described are not suited to protein 3D reconstruction given the different nature of distance constraints induced by the protein contact map. Several heuristic algorithms have been developed to address specifically the problem [[6-10], and [11]]. Routinely, most of the methods were also tested on randomly blurred contact maps derived from small sets of proteins (in the range of 20-30 chains) and no general conclusion was derived.

In order to address the problem of structure reconstruction we developed COMAR [12], and FT-COMAR 1.0 [13], both performing quite efficiently. With FT-COMAR we could analyze the reconstruction performance on a set of 100 protein contact maps containing random errors [13]. Recently a method focused on the search of the essential contacts in contact maps for protein 3D reconstruction. The method is however tested only on 12 proteins and this hampers again large scale statistical considerations [14].

In this paper we analyze the performances of FT-COMAR 2.0, a modified version of FT-COMAR 1.0 where reconstructed structures satisfy known protein constraints (available on the web [15]). Our tests are performed with a GRID technology on a much larger data set (1716 proteins) than in previous similar analysis from this group (100 proteins, [13]), and after introducing random blurring of the computed maps. By this, we derive some conclusions that may help future implementations of methods for 3D reconstruction. We investigate the reconstruction quality as dependent on the protein length, and on the four major SCOP classes. We also investigate the effect of three types of random errors, general and/or restricted to contacts and non-contacts. We find that the reconstruction quality decreases at increasing protein length and this is rather independent of the protein structural class. Furthermore we find that randomizing errors on the map is conducive to the same reconstruction performance that is obtained when errors are randomly restricted to non-contacts. On the contrary random errors on contacts are highly tolerated and up to 50% of contacts may be wrong without a great loss of 3D reconstruction quality (RMSD≤5 Å). We then address the question of how many correct contacts we need in order to reconstruct the protein and we find that only 25% of correct entries are sufficient to obtain a 3D structure with RMSD≤5 Å from the native one. This effect is independent of the protein length and indicates that FT-COMAR can correctly reconstruct the 3D structure even from a small fraction of correct contacts. Prompted by this finding we develop a filter procedure that when applied makes the protein reconstruction independent of the protein length as long as 10% of random errors is included in the map.

## Methods

### Data set

The protein dataset was selected from SCOP [16], release 1.67. We removed sequence redundancy by using BLAST [17] and retrieved from PDB only those complete structures whose resolution is <2.5 Å. Our final dataset consists of 1716 protein chains with sequence similarity <25%.

**Figure 1:**
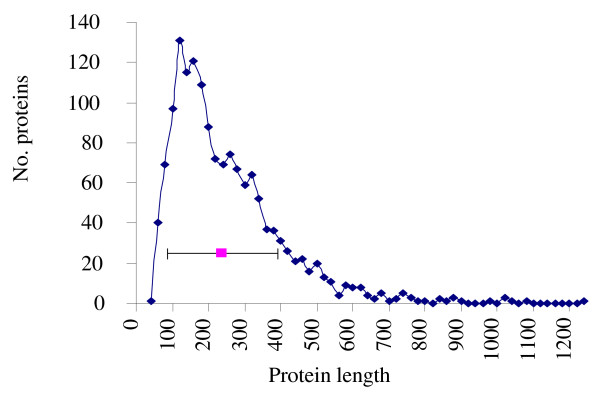
**Distribution of protein length**. The number of proteins (No. proteins) with a given length in our dataset is shown as a function of protein length (Protein length). The average length over all proteins is (239 ± 154) residues, as indicated.

The residue length distribution of the proteins in data set is shown in Figure 1. Noticeably most of the proteins have length ≤700 residues. The distribution of our dataset according to the SCOP classification is:

• 1362 mono domain proteins: 251 all Alpha, 286 all Beta, 376 Alpha/Beta, 332 Alpha+Beta; and 117 in other classes;

• 354 multi-domain proteins: 17 all Alpha, 42 all Beta, 46 Alpha/Beta, 39 Alpha+Beta; and 210 in other classes.

### Protein representation and contact maps

**Figure 2:**
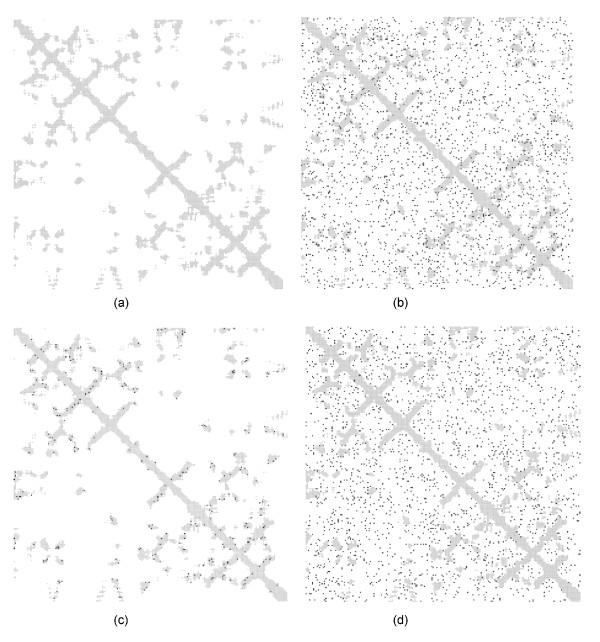
**Contact maps and random errors**. The contact map of the Asn102 mutant of trypsin (PDB code: 1trm chain A) is blurred with different types of random errors. Gray areas contain contacts (computed setting the threshold at 12 Å), white areas contain non-contact and black dots are errors. (a) The native map with 24753 residue pairs, of which 3595 are contacts, 21158 are non-contacts, and without random errors. (b) Err 5%. This map includes a number of random errors (1237) equal to 5% of total number of residue pairs. (c) Err-1 5%. In this map the total number of contacts is decreased by randomly substituting with non contacts (for a total of 179 non contacts corresponding to 5% of the original number of contacts). (d) Err-0 5%. In this map the total number of non contacts is decreased by randomly substituting with contacts (for a total of 1057 contacts corresponding to 5% of the original number of non contacts).

One of the most widely used representation of contact residues defines two residues *i *and *j *to be in contact when the Euclidean distance between their respective Cα atoms is below some given threshold *t*. Typical threshold values considered in literature vary between 7 and 12 Å. Threshold values equal to 7 and 8 Å minimize the distance between residue physical contacts and Cα contacts [18]. The residue-residue *contact map *of a protein is a two-dimensional approximation of the protein structure. Formally, a contact map of threshold *t *is the binary symmetric matrix *M *such that *M*_*ij *_= 1 if and only if the Euclidean distance between the Cα atoms of residues *i *and *j *is less than or equal to *t (*Figure 2a). As we showed in [12], the higher the threshold values the better is the 3D reconstruction; low threshold values often lead to very different structures (up to 40 Å RSMD) starting from the same contact map. Similarly to [13], in this work we adopt *t *= 12 Å for experiments where the effect of random errors is analyzed. To measure the similarity between two protein structures described by some set of coordinates *C*, *C' *∈ *R*^*3*^^×^^*n*^, we compute the Root Mean Square Deviation (RMSD), defined as:

(1) $$D_{k}=\sqrt{\frac{1}{n}\sum_{i=1}^{n}{\left(C'[i]-C_{k}[i]\right)}^{2}}$$

where *C*_*k *_∈ *R*^*3*^^×^^*n *^is obtained by rotating, translating, or mirroring the coordinates set *C*. Mirroring is needed since the native structure and its topological mirror share the same distance map and thus the same contact map. In this work we consider structures to be similar only when their RMSD value is ≤5 Å.

### Description of FT_COMAR 2.0

In this section we describe FT-COMAR 2.0, a fault tolerant version of COMAR generating 3D structures satisfying the backbone constrains.

**FT-COMAR**(*CM *∈ {-1,0,1}^*n*^^×^^*n*^, *t *∈ ***N***)

   //*Pre-processing phase: error filtering*

1: *CM' *← **FILTER**(*CM*)

   //*First phase: initial solution generation*

2: *C *← **FT-RANDOM-PREDICT**(*CM', t*)

   //*Second phase: refinement*

3: *C *← **FT-CORRECT**(*CM', C, t*)

4: *set ε to a strictly positive value*

5: **while ***C is not a Cα trace consistent with CM' ***and **ε *> 0 ***do**

6: *C *← **FT-PERTURBATE**(*CM', C, t*, ε)

7: *C *← **FT-CORRECT**(*CM', C, t*)

8: *decrement slightly ε*

9: **if ***C is not a Cα trace ***then ***C *← **Cα-TRACE**(*CM', C, t*)

10: **return ***C*

FT-COMAR consists of three phases. In the pre-processing phase, the input contact map is scanned with a filtering procedure (**FILTER**) in order to mark the unsafe entries. The marked entries will then be ignored in the next computations (**FT-RANDOM-PREDICT**, **FT-PERTURBATE **and **FT-CORRECT**). In the first phase (Phase 1), the algorithm generates a random initial set of 3D coordinates *C *∈ *R*^3 ×^^*n *^(**RANDOM-PREDICT**) that is the starting point for the refinement procedure. In Phase 2 the algorithm iteratively applies two local correction/perturbation techniques to the current set of coordinates, **FT-CORRECT **and **FT-PERTURBATE**. This procedure refines the initial set of coordinates and eventually leads to a new set of coordinates that are completely or almost completely consistent with the given contact map. The refinement continues until the set of coordinates satisfies the protein constraints provided by the input contact map or until a control parameter *ε *becomes 0. The control parameter *ε *has an initial positive value and it is iteratively decremented after some refinement steps.

As a final check, the **Cα-TRACE **function ensures that the reconstructed structure satisfies the backbone constrains, namely the distance between consecutive coordinates, set between 3.5 and 4 Å, and the minimum distance between any pair of coordinates, set to 3.5 Å. The **FILTER **function identifies unsafe areas of the contact map. The functions **FT-RANDOM-PREDICT**, **FT-CORRECT **and **FT-PERTURBATE **are similar to the non fault tolerant version, with the only difference of neglecting entries of the contact map labelled as unsafe. **FT-RANDOM-PREDICT **computes the initial solution. When fragments of the protein demonstrate a high degree of independence with respect to mutual interactions, **FT-RANDOM-PREDICT **splits the initial contact map into submatrices,. Then a set of coordinates is separately generated for each sub matrix with an embedding algorithm [3]. The sets of coordinates are then merged to give the initial solution. **FT-CORRECT **moves residues in the reconstructed 3D structure in order to decrease the difference between entries of the computed and input contact maps while preserving identical values. Concomitantly with **FT-CORRECT**, **FT-PERTURBATE **perturbs the residue position for optimising the overlap of contact maps. Details on these functions can be found in [12].

In the following we describe **FILTER **and **Cα-TRACE **as a new development. **FILTER **searches input contact maps for 'unsafe' areas, namely false entries due to noise. This is implemented by assuming that two residues i,j are in contact if and only if they share a high number of neighbors, i.e. there is a high number of residues which are in contact with both i and j. In our dataset, at the selected contact threshold (12 Å, section 2.2), only 6% of residues which are in contact share less than 10 neighbors and just the 0.7% of residues which are not in contact share >18 neighbors. Thus our filtering procedure marks contact C [i, j] as unsafe (setting C [i, j] to -1) if:

• C [*i*, *j*] = 1 (*i *and *j *are in contact) and *i*, *j *share <10 neighbours, i.e. residue *i *is in contact with <10 residues which are in contact also with residue *j*;

• C [*i*, *j*] = 0 (*i *and *j *are not in contact) and *i*, *j *share >18 neighbours, i.e. residue *i *is in contact with >18 residues which are in contacts also with residue *j*.

**FILTER **output is the contact map with unsafe areas set to -1. These entries are then neglected by FT-COMAR.

The **Cα-TRACE **function changes a given set of coordinates to satisfy the following constraints as derived from the Cα protein representation:

• the distance between consecutive coordinates *i*,*i+1 *is between 3.5 and 4 Å;

• the distance between any pair of coordinates *i,j *is ≥3.5 Å.

The coordinate refinement is obtained with a correction/perturbation cycle [similarly to the refinement phase of FT-COMAR (section 2.3)].

**Cα-TRACE (***CM *∈ {-1,0,1}^*n*^^×^^*n*^, *C *∈ ***R***^*3*^^×^^*n*^*, t *∈ ***N***)

1: *set *ε *to a strictly positive value*

2: **while ***C is not a Cα trace consistent with CM ***and **ε *> 0 ***do**

3:   *C *← **FT-PERTURBATE-TRACE**(*CM, C, t*, ε)

4:   *C *← **FT-CORRECT-TRACE**(*CM, C, t*)

5:   *decrement slightly ε*

6: **if ***C is not a Cα trace ***then Cα-TRACE-FIX**(*C, t*)

7: **return ***C*

Here **FT-PERTURBATE-TRACE **and **FT-CORRECT-TRACE **are similar to **FT-CORRECT **and **FT-PERTURBATE **with the only addition of the **Cα-TRACE **constraints. **FT-CORRECT-TRACE **moves residues and **FT-PERTURBATE-TRACE **refines their mobility. When after refinement (lines 1-5 of **Cα-TRACE**) the set of coordinates *C *is not a *Cα *trace, the function **Cα-TRACE-FIX **imposes the **Cα-TRACE **constraints neglecting the original contact map. This is obtained by running **Cα-TRACE **with an "unsafe" contact map (all entries set to -1).

### Introducing random errors in real contact maps

To evaluate fault tolerance of FT-COMAR to *white noise *(i.e. random errors) we introduce three types of random errors:

• **Err**. A random error is generated by flipping a random entry of the native contact map (Figure 2b). To introduce *x*% errors we generate *x *errors for each 100 couples of residues and the total number of errors is:

(2) $$\frac{x}{100}\frac{n(n-1)}{2}$$

• **Err-1 **(errors on contacts). The entry of the contact map is flipped only if it is a contact (Figure 2c). Here *x*% errors indicate that the total number of errors is:

(3) $$\left(\frac{x}{100}.\#contacts\right)$$

• **Err-0 **(errors on non-contacts). Errors are generated as before by changing entries in the contact map only for non contacts (Figure 2d). Here *x*% errors indicate that the total number of errors is:

(4) $$\frac{x}{100}\left(\frac{n(n-1)}{2}-\#contacts\right)$$

where *n *is the protein length.

We generate 10 (distinct) perturbed maps by introducing x% random errors on the native map and run our algorithm, partially randomized, 10 times on each map. By this in order to test the reconstruction tolerance in presence of x% random errors for every native contact map, we generate 10 perturbed contact maps and compute 10 reconstructions for each map, for a total of 100 runs.

### Computational environment

Testing FT-COMAR is computationally expensive since it requires several applications that must be run to introduce errors in contact maps, compute the reconstruction and evaluate the performances. Each execution is repeated 100 times, as described in section 2.3, for a total of 12,154,234 jobs. This is a typical example of parameter sweep application (PSA), i.e. it consists of many loosely-coupled tasks that can be executed in parallel [19,20]. The single execution runs in a time ranging from micro seconds to several minutes depending on the protein length and on the percentage of errors introduced. Here the whole experiment was run by using the LIBI Grid PSE [21]. The average number of jobs running concurrently over the EGEE and SPACI Grid infrastructures was about 120 with a total of 4,500 different worker nodes. By this the execution time was greatly reduced from 34.16 years on a typical pc to about three months.

## Results and Discussion

### Protein structure reconstruction from contact maps with white noise

**Figure 3:**
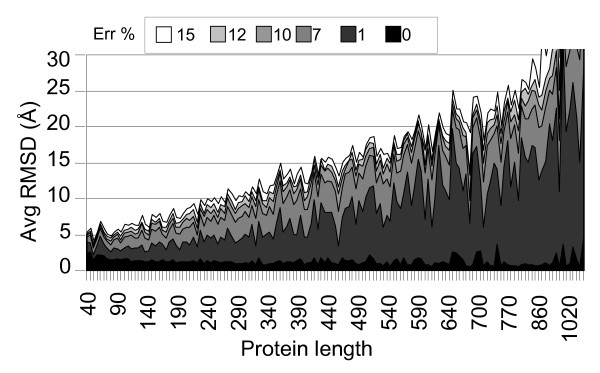
**Reconstruction of proteins from contact maps with random errors**. The average RMSD (Avg RMSD) value of the native structure to the corresponding ones reconstructed from blurred contact maps is shown as a function of protein length (Protein length), and at increasing percentage of added random errors (Err %, from 0 up to 15%). The number of errors relative to the same percentage increases with protein size: e.g. 10% of random errors for a protein with length 100 (residues) corresponds to 450 errors; 1% of random errors for a protein with length 400 corresponds to 798 errors. 90,000 contact maps are analyzed.

The performance of FT-COMAR is analyzed by introducing white noise in the contact maps. The results are obtained on a set of non-homologous proteins which is orders of magnitude larger than any set adopted so far [6, 7, 8, 9, 10, and 11]. For each protein and each percentage of random errors 10 different noisy contact maps are generated. Then, for each noisy contact map we performed 10 different reconstructions. Results in Figure 3 are obtained by averaging RMSD over about 1,000,000 reconstructions. The results indicate that the reconstruction quality decreases at increasing percentage of random errors and at increasing protein length (Figure 3). Considering the length distribution of the protein set we can conclude that FT-COMAR safely reconstruct proteins of any length starting from contact maps without errors. When white noise is incrementally added, proteins with length ≤ 350 residues are safely reconstructed (RSMD≤5 Å) provided that blurring affects 1% of the contact map. At increasing percentage of added random errors, native protein reconstruction fails: the longer the proteins the lower is the percentage of tolerated random errors (Figure 3). This is due to the fact that at a fixed percentage the number of errors increases with protein size (for instance 10% of random flips introduced on a 100 residue-long protein correspond to 450 errors added to its contact map, while 1% random errors on a protein of 400 residues amount to 798 contact map errors).

**Figure 4:**
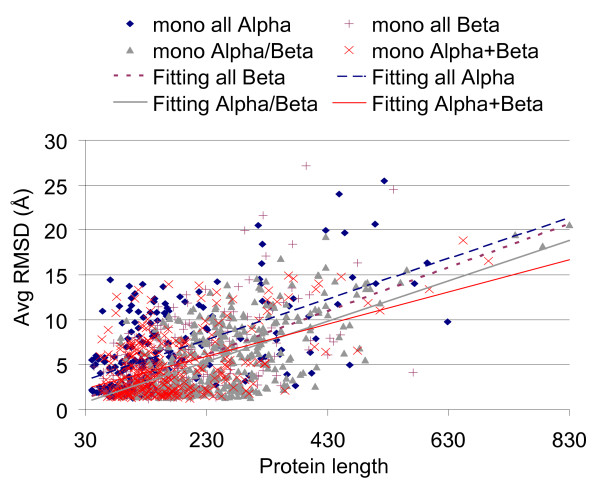
**Reconstruction of SCOP monodomain proteins from contact maps with random errors**. The average RMSD (Avg RMSD) value between the native structure and the corresponding ones reconstructed from contact maps blurred with 5% random errors is plotted as a function of the protein length (Protein length). Results are plotted by distinguishing the different SCOP structural classes, as indicated. Each dot represents the average quality of the reconstructions of a given protein. Only mono-domain proteins of the four major SCOP classes are considered (see section 2.1). For each SCOP structural class, a linear least square fitting curve of the data is computed.

**Figure 5:**
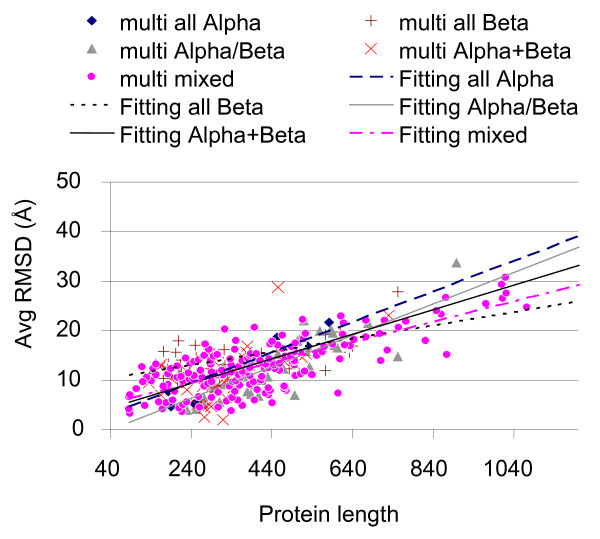
**Reconstruction of SCOP multidomain proteins from contact maps with random errors**. The average RMSD (Avg RMSD) value between the native structure and the corresponding ones reconstructed from contact maps blurred with 5% random errors is plotted as a function of the protein length (Protein length). Here only multi-domain proteins of the four major SCOP classes are considered (see section 2.1). Legend is as in Figure 4.

Reconstruction is also evaluated as function of protein folds and for sake of clarity we separately consider monodomain and multidomain proteins that are listed according the four major SCOP structural classes, respectively. In all cases contact maps were blurred with 5% random entries. The reconstruction quality is decreasing more as a function of the protein length than considering the SCOP classes, and this is so both for monodomain and multidomain proteins (Figure 4 and 5). On average, FT-COMAR performances are worse on all-alpha proteins. We find that proteins for which the contact map is not informative, i.e. the native contact map corresponds to highly different 3D structures, are abundant only in the all-alpha protein set [12]. This behaviour is possibly due to the on average lower content of long range contacts in all-alpha protein contact maps than in the other proteins.

### Protein structure reconstruction from contact maps as a function of the white noise type

**Figure 6:**
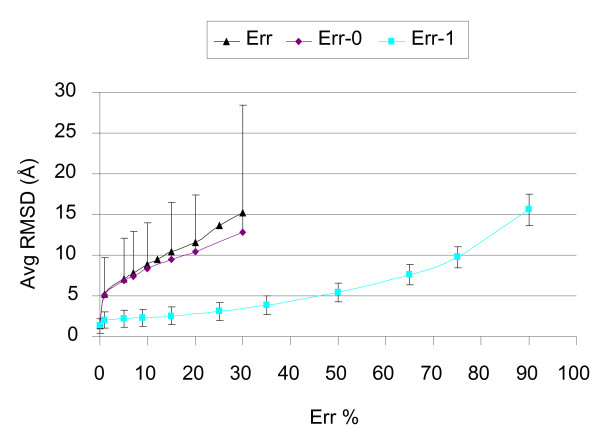
**Structure reconstruction from contact maps with different types of random errors**. The average RMSD (Avg RMSD) value of the native to the corresponding protein structures reconstructed from blurred contact maps is plotted as a function of the percentage of random errors in the contact maps (Err %). Contact maps are blurred with three types of random errors: errors on both contacts and non contacts (Err), errors on contacts (Err-1) and errors on non-contacts (Err-0). For clarity reasons, error bars representing standard deviation are plotted only for Err-1 and for Err-0 (with only the upper bar). Each dot in the plot is the average value over 1716 proteins.

FT-COMAR reconstructs contact maps containing different types of random errors (see section 2.3). Our results (Figure 6) indicate that reconstruction within the limiting threshold value (RSMD≤5 Å) is tolerating random noise on contacts more than on non-contacts. The fault tolerance pattern of reconstruction as a function of added random noise to non-contacts (Err-0) overlaps that of all entries (Err) (Figure 6). Reconstruction is much more tolerant to white noise when it affects only contacts (Err-1). In Figure 6, the average RMSD value of contact maps with 50% randomly flipped contacts (from contact to non contact) is about 5 Å. This value is quite similar to that of contact maps where 1% of the non-contacts randomly flips to contacts. The high standard deviation indicates that for each percentage of Err-0 and Err in a contact map both high and low quality reconstructions are obtained and this depends mostly on the effect of protein length, as shown above. On the contrary, for the Err-1 experiments the obtained standard deviation is small, indicating that when contacts randomly flip to non contacts, reconstruction quality is independent of protein length. Furthermore, if we consider that the number of contacts in a typical contact map corresponds to about 5% of the entries we can estimate that the number of errors in 1% of the non-contacts roughly correspond to 20% of the contact entries. Even in this case, considering the sheer number of errors instead of the percentage, when we restrict errors to contacts (Err-1) we obtain more accurate reconstructions. These findings confirm that contact maps with errors on contacts (under predictions) can be used to reconstruct the 3D protein structure more accurately than contact maps with errors on non-contacts (over predictions).

### Reconstruction of contact maps as function of its partial deletion

**Figure 7:**
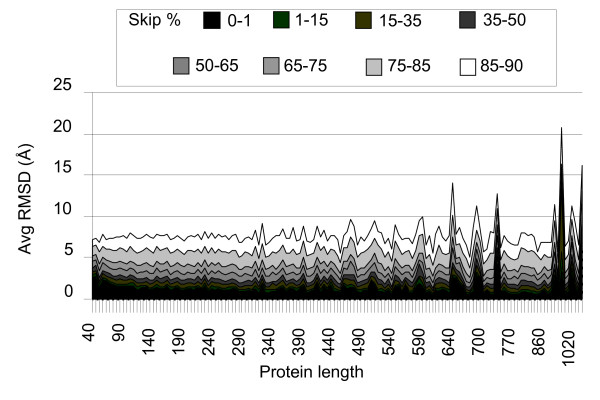
**Structure reconstruction after random partial deletion of the contact map**. The average RMSD (Avg RMSD) value of the native and corresponding protein structures reconstructed from incomplete maps is plotted as a function of the protein length (Protein length) and increasing percentage of random deletion of the contact map (Skip %). The number of deleted entries relative to the same percentage of deletion (Skip %) increases at increasing protein length.

**Figure 8:**
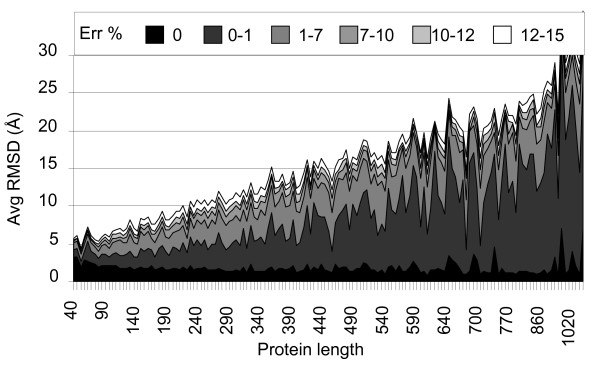
**Structure reconstruction after partial deletion of contact maps and with random errors**. The average RMSD (Avg RMSD) between the native and corresponding protein structures reconstructed from contact maps with 25% of random deletion and increasing amount of random errors (Err%, from 0 to 15%) is plotted as a function of protein length (Protein length).

In order to quantify the amount of information needed to obtain a high quality reconstruction we randomly removed different amounts of contact map entries. Adopting this procedure, we verify that on average FT-COMAR can tolerate up to 75% randomly skipped entries (Figure 7) when reconstructing protein structure in the whole interval of lengths considered. As a second step we analyze the effect of deleting entries on blurred contact maps. As an example we show the results in Figure 8. Here 25% of the entries on the noisy contact maps were removed and the results indicate that overall reconstruction occurs with the performances already described in Figure 3. This finding suggests that a large fraction of the map entries can be deleted in order to remove noise without affecting the reconstruction performance.

### Reconstruction of contact maps as function of pre-filtered white noise

**Figure 9:**
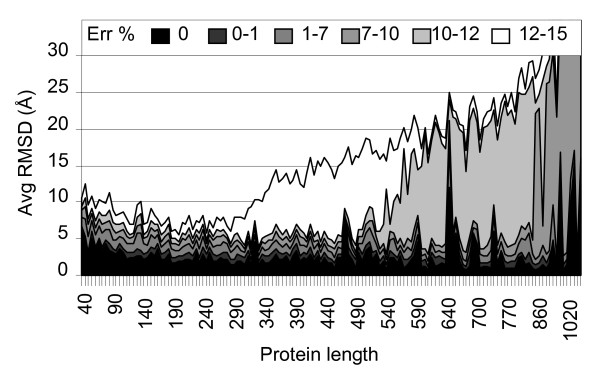
**Structure reconstruction after pre-filtering of contact maps with random errors**. The average RMSD (Avg RMSD) of the native and corresponding protein structures is evaluated upon reconstruction after pre-filtering of contact maps containing increasing amounts of random errors (Err %) and is plotted as a function of protein length (Protein length). The filtering procedure checks for neighbouring properties (see The description of FT_COMAR 2.0, Methods).

The high tolerance of FT-COMAR to entry deletion is exploited by implementing a method suited to selectively removing errors from a noisy contact map. Taking into consideration that on average contacts tend to cluster [13], we designed a simple pre-filtering procedure which processes entries by sorting them in relation to the number of common contacting residues (see Methods). The following rules were implemented: 1) a contact between two residues *i *and *j *is deleted if there are too few common contacting residues; 2) a non-contact is deleted if there are too many common contacting residues. The results obtained when this pre-processing is applied are reported in Figure 9. It appears that the reconstruction quality increases for medium size and long proteins up to 10% random errors. Furthermore, the reconstruction quality becomes nearly independent of the protein length up to 8-10% random errors.

### Computing time

**Figure 10:**
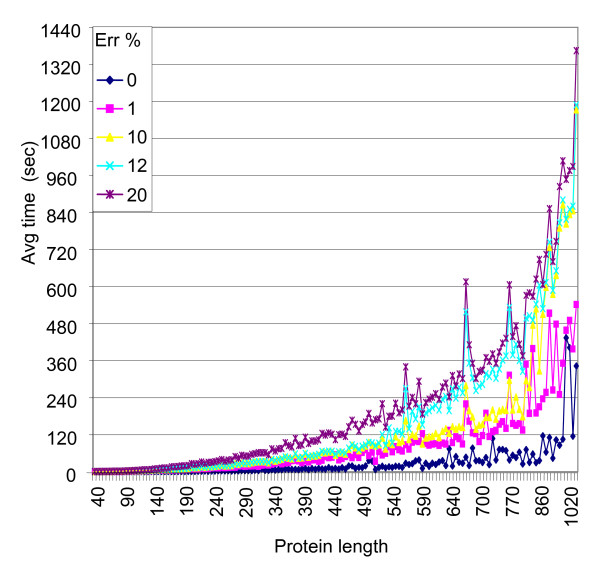
**Computational time for protein structure reconstruction**. The average time (Avg time) needed to reconstruct structures of proteins of a given length from contact maps containing increasing percentages of random errors is plotted as a function of protein length (Protein length). A filter checking neighbouring properties is adopted during reconstruction, as in Figure 9.

FT-COMAR is suited for large-scale experiments because it is a reasonable fast algorithm, as shown in Figure 10. Here, the time needed to reconstruct the noisy contact maps with the filter procedure is shown as a function of the protein length. As expected, the running times get worse at increasing protein size and increasing percentages of errors in the map, ranging from a fraction of second for short proteins to nearly half a hour for long proteins. It is worth noticing that contact maps that are better reconstructed require less running time.

## Conclusions

Reconstruction of contact maps is a necessary step of 3D protein reconstruction. The step is particularly relevant when contact maps are predicted. Presently the prediction quality of contact maps is still too low to allow protein reconstruction and this has been discussed elsewhere [15]. In this work we focus on the effect of white noise on contact map reconstruction with the specific aim of setting some constraints for future developments. For this reason we undertook a large scale analysis of the effect of random noise on the reconstruction of contact map with our FT-COMAR. Reconstruction quality decreases at increasing protein length and it is rather independent of the protein structural class, with the exclusion of all-alpha proteins that on average are the most difficult to reconstruct. This can be reconciled with the suggestion that in contact maps long range contacts play a critical role in 3D reconstruction [1,18] and that all alpha proteins are endowed with less long range contacts than the other SCOP classes.

The large scale analysis that allows a more accurate statistics than before indicates also that 25% of the randomly selected entries of the native contact map is enough to correctly reconstruct the protein structure. Considering that introducing random errors quickly degrades the quality of reconstruction and that this is not due to random flipping of contacts into non-contacts we conclude that the correctness of contacts in the map is more important than their relative abundance. Therefore our large-scale effort validates the concept that wrong contacts make the reconstruction more problematic than missed contacts. Essential contacts for protein reconstruction were described before [14]. Also in our hand and for FT_COMAR, few key contacts are more conducive to the real/close-to-the-real protein structure than many noisy contacts. Prompted by this, we developed a simple filtering procedure. Its application that labels "unsafe" certain blurred areas of the map, greatly improves the quality of reconstructed structures even for long protein chains. All together these findings are landmarks to be considered in developing future 3D reconstruction tools and also predictors of contact maps.

## Competing interests

The authors declare that they have no competing interests.

## Authors' contributions

MV developed and implemented FT-COMAR, prepared testing programs, checked them on a reduced dataset, and drafted the manuscript. LM mainly designed FT-COMAR. PDL contributed in improving and reengineering FT-COMAR. PF contributed to the analysis of results. MM and GA executed the tests on the large scale dataset managing the LIBI Grid PSE. RC coordinated the whole project, contributed to the analysis of results and to the final version of the manuscript. All authors read and approved the final manuscript.
